# *Lactobacillus* delivery of bioactive interleukin-22

**DOI:** 10.1186/s12934-017-0762-1

**Published:** 2017-08-23

**Authors:** Yin Lin, Kasper Krogh-Andersen, Lennart Hammarström, Harold Marcotte

**Affiliations:** Division of Clinical Immunology and Transfusion Medicine, Department of Laboratory Medicine, Karolinska Institutet at Karolinska University Hospital Huddinge, 141 86 Stockholm, Sweden

**Keywords:** *Lactobacillus paracasei* BL23, Mouse interleukin-22, GVHD

## Abstract

**Background:**

Interleukin-22 (IL-22) plays a prominent role in epithelial regeneration and dampening of chronic inflammatory responses by protecting intestinal stem cells from immune-mediated tissue damage. IL-22 has a considerable therapeutic potential in graft-versus-host disease (GVHD), which is a frequent and challenging complication following allogeneic stem cell transplantation. The aim of our study was to engineer *Lactobacillus* for delivery of IL-22 directly to the intestinal mucosa as a new therapeutic strategy for GVHD.

**Results:**

The secretion and surface anchoring of mouse IL-22 by *Lactobacillus paracasei* BL23 was demonstrated by Western blot and flow cytometry. Both secreted and anchored mouse IL-22 produced by *Lactobacillus* was biologically active, as determined by its ability to induce IL-10 secretion in the Colo 205 human colon cancer cell line.

**Conclusions:**

We have demonstrated the secretion and surface anchoring of bioactive IL-22 by *Lactobacillus.* Our results suggest that IL-22 expressing lactobacilli may potentially be a useful mucosal therapeutic agent for the treatment of GVHD, provided that chromosomal integration of the IL-22 expression cassettes can be achieved.

**Electronic supplementary material:**

The online version of this article (doi:10.1186/s12934-017-0762-1) contains supplementary material, which is available to authorized users.

## Background

Interleukin-22 (IL-22) is a member of the IL-10 family of cytokines, expressed predominantly by subsets of innate lymphoid cells (ILCs) and activated T cells, including T helper 1 (TH1) cells, TH17 cells and TH22 cells [1, 2]. IL-22 can be recognized by a heterodimeric receptor complex that consists of two transmembrane subunits: IL-22R1 and IL-10R2 [3]. The binding of IL-22 to its receptor activates the JAK/STAT and MAPK signaling pathway [4, 5], resulting in gene expression or repression. Since the IL-10R2 is shared by five cytokines (IL-10, IL-22, IL-26, IL-28, and IL-29) and is widely expressed in most cells [6], the expression of the IL-22R1 determines whether a cell is the target of IL-22. The lack of expression of IL-22R1 in all immune cells indicates that IL-22 does not directly regulate the function of the immune system [7]. The targets of this cytokine are mostly non-hematopoietic epithelial and stromal cells [2, 8] in organ tissues including intestines, lung, liver, kidney, thymus, pancreas, and skin [1]. IL-22 can induce the production of antibacterial peptides from epithelial cells and selected chemokines in specific tissues, where it can promote epithelial cell survival and proliferation, and play a role in tissue regeneration and protect against damage induced by chronic inflammation [1, 9–11]. Local IL-22 gene delivery has been shown to lead to rapid attenuation of intestinal inflammation in the colon in a Th2-mediated chronic colitis mouse model [12]. Thus, IL-22 is an attractive and promising target for inflammatory bowel disease (IBD) therapy [13].

Graft-versus-host disease (GVHD) is a common complication that can occur after an allogeneic tissue transplantation in which the newly transplanted material attacks the cells of recipient [14]. Following haematopoietic stem-cell transplantation, acute GVHD occurs when graft-derived T cells are activated against antigens from the recipient involving mainly the skin, gastrointestinal (GI) tract and liver [15, 16]. Acute GVHD of the GI tract, which is histologically similar to IBD [17], is often severe and is a significant cause of transplant-associated morbidity [16]. Rorγt^+^ IL-23-responsive innate lymphoid cells (ILCs), which are present in gut cryptopatches, are a major source of IL**-**22 [18]. Previous studies have shown that IL-22 deficiency in recipient mice leads to increased intestinal GVHD pathology and accelerated mortality [16]. Treatment with recombinant mouse IL-22 via intraperitoneal (i.p.) injection after mouse allogeneic bone marrow transplantation enhanced the recovery of intestinal stem cells (ISCs), increased epithelial regeneration and reduced intestinal pathology and mortality from GVHD [19]. The addition of recombinant mouse IL-22 to both mouse and human small intestinal organoid cultures results in larger organoids than without IL-22 and directly increases proliferation and expansion of ISCs (independent of Paneth cells) [19].

Due to the restricted distribution of the IL-22R, and given the lack of this receptor on immune cells, IL-22 could be used as a relatively safe way to treat GVHD without causing immune-related side effects [1]. An oral formulation could be a potential way to deliver IL-22 directly to intestinal GVHD lesions.

*Lactococcus lactis* has previously been used to secrete IL-22 under a nisin-inducible promoter [20]. However, *Lactococcus* does not colonize the intestinal tract and the inducible promoter is not suitable for gastrointestinal delivery. In contrast, lactobacilli are normal residents of the GI tract of animals and humans [21]. They have historically been used in food preservation and are formally recognized as “generally recognized as safe” (GRAS) organisms. Selected probiotic *Lactobacillus* strains can colonize the gastrointestinal tract for week [22] where they may constitutively produce the desired peptide, reducing their exposure to gastric acid, bile and digestive enzymes. Antibody fragments and incretin hormone have previously been delivered by lactobacilli to treat viral infections or diabetes [23, 24]. A major shift in the composition of the intestinal microbiota as well as increased permeability of the intestinal mucosa and bacterial translocation is associated with aggravation of GVHD symptoms [25–27]. Reintroducion of *L. johnsonii* in a mouse model was shown to alleviate GVHD lethality and pathology probably due to the prevention of *Enterococcus* expansion which may otherwise exacerbate GVHD-associated intestinal inflammation [25]. Genetically engineered *Lactobacillus* could be used to deliver IL-22 directly to the intestinal GVHD lesions, providing a continuous supply of bioactive IL-22 during disease progression. The secreted IL-22 or lactobacilli expressing surface anchored IL-22 could cross the damaged mucosa and bind to the IL-22 receptor on the basolateral surface of epithelial cells.

In this study, we engineered *Lactobacillus paracasei* BL23 to express mouse IL-22 constitutively in both secreted and cell wall-anchored forms. The *Lactobacillus* produced mouse IL-22 was proven to be biologically active in an in vitro cell culture model.

## Methods

### Bacterial strains and growth conditions

*Lactobacillus paracasei* BL23 (previously named *L. casei* 393 pLZ15^−^) [28, 29] was inoculated in liquid MRS medium (Difco, Sparks, MD) at OD_600_ = 0.08 from the overnight culture and grown at 37 °C statically to OD_600_ = 1.0 (2 × 10^8^ cfu/ml) or anaerobically (BD—GasPak EZ, Sparks, MD) on MRS-agar plates. For improved IL-22 stability in the culture medium, the initial pH of MRS medium was adjusted to 8.5 by the addition of an appropriate amount of sodium hydroxide, and then filter-sterilized (0.22 μM). *E. coli* DH5α (Invitrogen, Carlsbad, CA) was used as a general cloning host and cultured in Luria–Bertani broth in an orbital shaker or on LB-agar plates at 37 °C. When required, antibiotics were added as follows: 5 µg/ml chloramphenicol for *L. paracasei* BL23, and 100 µg/ml ampicillin for *E. coli* DH5α.

### Construction of mouse IL-22 expression vectors

**Fig. 1:**
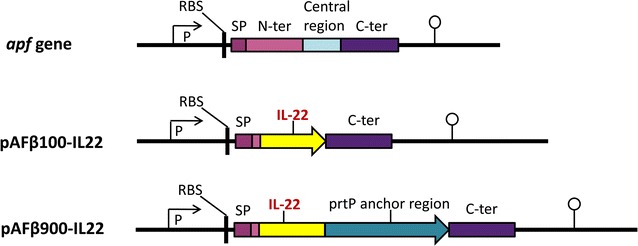
Expression cassettes of IL-22. SP, signal peptide of *apf* gene (including its start codon); RBS, ribosomal binding site; P, *apf* promoter; C-ter, C-terminal part of *apf* gene; mouse IL-22 or prtP translational stop codon (both indicated with an *arrowhead*) and the transcription terminator of *apf* gene (indicated with a *lollipop*). The C-terminal part of the *apf* gene is not translated

Two expression cassettes were generated by fusing the mouse IL-22 gene with the promoter region and part of the gene encoding the APF protein of *L. crispatus* M247 (Fig. 1) [30, 31]. The two expression cassettes differ only by the fusion of the anchored region of the prtP gene on the C-terminal of the IL-22 gene, which gives covalent cell wall binding of the recombinant cytokine on the bacterial surface.

The plasmid construct is similar to the previously published pAF100 and pAF900 [31]. Briefly, two fragments of *apf* gene were amplified using *L. crispatus* M247 genomic DNA as a template: fragment 1 containing the promoter region, the signal peptide and the first four amino acids of the N-terminal domain of the *apf* gene was amplified using primers APFSalS and APFBamAS; fragment 2 containing the C-terminal domain and the terminator region of the *apf* gene was amplified using primers APFSacS and EcoAS2. Fragment 1 and 2 were cloned into the plasmid pIAβ8 [32] between *Sal*I and *Bam*HI and between the *Sac*I and *Eco*RI restriction sites, respectively, resulting in pIAβ100. The prtP anchor region encoding gene was amplified from a pLP401-scFv-long anchor plasmid [33] using the primers prtPSacIS and prtPSacIAS and cloned in the *Sac*I restriction site of pIAβ100 generating pIAβ900. The primers used for amplification are shown in Additional file 1: Table S1.

The mouse IL-22 gene was produced as a synthetic gene with codons optimized for expression in *L. paracasei* (GenScript, Piscataway, NJ). The synthetic genes (Additional file 1: Table S1), flanked by an upstream *Bam*HI and a downstream *Sac*I restriction site, were cloned between the *Bam*HI and *Sac*I sites in the *Lactobacillus* expression vectors pAFβ100 and pAFβ900 [31]. The two generated plasmids, pAFβ100-IL22 and pAFβ900-IL22, mediate secretion and surface anchoring of mouse IL-22, respectively. The plasmids were first transformed into *E. coli* DH5α and the expression cassettes were verified by DNA sequencing. The plasmids were subsequently transformed into *L. paracasei* BL23 by electroporation as previously described [33, 34], generating *Lactobacillus* strains expressing IL-22 which were denoted Lp pAFβ100-IL22 or Lp pAFβ900-IL22.

### Western blot analysis

The expression of mouse IL-22 from *Lactobacillus* was confirmed by Western blot. The bacterial cultures were spun down when OD_600_ reached 1.0 and the supernatant and bacterial pellets were separated for protein extraction. The supernatant was filter-sterilized and the pH adjusted to 7.0. Then, 100 μl of supernatant was mixed with an equal volume of 2× Laemmli buffer (Bio-Rad) and boiled for 5 min. The bacterial pellets from 1 ml cultures were washed twice with PBS and resuspended in 100 μl 2× Laemmli sample buffer and boiled for 10 min. The cell debris was removed by centrifugation at 16,100×*g* and the supernatant, denoted as cell extract, was retained. Ten ng of commercial recombinant mouse IL-22 (R & D Systems) was used as a positive control, and 20 μl of the supernatant or cell extract sample were run on SDS-PAGE gel and transferred onto nitrocellulose membrane (Hybond-ECL, GE Healthcare, UK). A biotinylated goat anti-mouse IL-22 antibody (R & D Systems) was used as the primary antibody at 0.2 μg/ml. Streptavidin-HRP (SAv-HRP) (BD Pharmingen, USA) was used as secondary antibody at a 1:2000 dilution. Furthermore, the amount of IL-22 produced in the supernatant of *Lactobacillus* (Lp pAFβ100-IL22) cultures was quantified by Western blot densitometry and compared to commercial recombinant IL-22 protein.

### Flow cytometric analysis

Cell wall display of mouse IL-22 on the surface of *Lactobacillus* was confirmed by flow cytometric analysis. Fifty μl of *Lactobacillus* (Lp pAFβ900-IL22) cultures grown in MRS to an OD_600_ of 1.0 were pelleted by centrifugation (3000×*g* for 10 min) and washed twice in PBS. Bacteria were incubated with biotinylated goat anti-mouse IL-22 antibody (1 μg/ml) for 30 min on ice. After two PBS washes, the cells were incubated with FITC conjugated streptavidin (BioLegend, San Diego, CA, 1:500 dilution) on ice for 30 min in the dark. The antibody and conjugated streptavidin were diluted in PBS containing 1% BSA. After washing three times with PBS, samples were resuspended and fixed in 400 μl 1% paraformaldehyde and analyzed using a FACS Calibur machine (Becton–Dickinson, Franklin Lakes, NJ).

### IL-22 biological activity assay

The activity of *Lactobacillus* produced mouse IL-22 was measured by its ability to induce IL-10 secretion in the Colo 205 human colon carcinoma cell line [35]. Colo 205 cells were maintained in RPMI-1640 supplemented with 10% FCS, 100 U/ml penicillin and 100 μg/ml streptomycin. Cells were grown loosely attached and in suspension in flask until confluent, and were seeded in 6-well plates at a density of 1 × 10^6^ cells/well several hours before addition of IL-22.

Two strains of mouse IL-22 expressing lactobacilli were tested in the experiment: Lp pAFβ100-IL22 secreting IL-22 and Lp pAFβ900-IL22 producing surface anchored IL-22. The wild type *L. paracasei* BL23 strain was used as a negative control. The supernatants of Lp pAFβ100-IL22 and wild type *L. paracasei* BL23 from 10 ml MRS culture (initial pH 8.5) were harvested at OD_600_ = 0.8 and adjusted to pH 7.0 before use. The bacteria were killed with UV before use in the assay in order to prevent acidification of the culture medium. The bacterial pellets of Lp pAFβ900-IL22 and wild type *L. paracasei* BL23 were washed twice with PBS, resuspended in PBS and treated with UV light using a cross linker (for 2 min at 120 mJ/cm^2^). Commercial recombinant mouse IL-22 (R & D Systems) (0.02–25 ng/ml), dilution of bacterial culture supernatant (1/25, 1/125, 1/625, 1/3125) or killed bacteria (5 × 10^9^, 1 × 10^9^, 5 × 10^8^, 1 × 10^8^) in RPMI-1640 were added to Colo 205 cells in a total volume of 2 ml in 6-well plates. The cells were incubated at 37 °C for 22 h in a humidified incubator containing 5% CO_2_. After incubation, the supernatant was collected from each well and tested for human IL-10 concentration by specific sandwich ELISA.

### ELISA

ELISA was used to quantify the expression level of IL-22 in the supernatant of Lp pAFβ100-IL22. Flat-bottom, 96-well EIA/RIA plates (Costar) were coated with goat anti-mouse IL-22 antibody (R & D Systems) and incubated overnight at 4 °C. After washing with PBS containing 0.05% Tween 20 (PBST) and blocking with 1% BSA in PBST, serial dilutions of the supernatant (1/10 to 1/1280) from Lp pAFβ100-IL22 cultures and wild type *L. paracasei* BL23 (negative control) were added and incubated at room temperature for 2 h. Recombinant mouse IL-22 (R & D Systems) was used to create a standard curve at concentrations of 500 ng/ml to 244 pg/ml. Plates were washed with PBST and a biotinylated goat anti-mouse IL-22 antibody (0.2 μg/ml, R & D Systems) was added and incubated at room temperature for 1 h, plates were subsequently washed with PBST and streptavidin–alkaline phosphatase (1/4000 dilution, BD Pharmingen) was used for detection, following incubation at room temperature for 1 h. Diethanolamine buffer (1 M, pH 10.0) containing 1 mg/ml of pNPP (Sigma-Aldrich) was added to the wells. After 30 min incubation, absorbance was read at 405 nm in a Varioskan Flash microplate reader (Thermo Electron Corporation, Vantaa, Finland).

The amount of IL-10 in the cell-free medium from the Colo 205 cell stimulation experiments was also determined by ELISA. Goat anti-human IL-10 (1 μg/ml, Sigma) was used to coat the plate and recombinant human IL-10 (BD Pharmingen) was used as a standard at concentrations of 500 ng/ml to 244 pg/ml. Biotinylated goat anti-human IL-10 antibody (1 μg/ml, R & D Systems) was used as the primary antibody and streptavidin- alkaline phosphatase (1/4000 dilution, BD Pharmingen) was used for detection.

## Results and discussion

### Construction of *Lactobacillus* constitutively expressing mouse IL-22

Two different expression plasmids were constructed for production of mouse IL-22 secreted in the supernatant (pAFβ100-IL22) or anchored on the cell surface (pAFβ900-IL22) (Fig. 1). These two expression plasmids are very similar to our previously published pAF100 and pAF900 plasmids [31]: both are regulated by the constitutive promoter of the *aggregation promoting factor* (*apf*) gene from *Lactobacillus crispatus* M247 and secretion is mediated by the signal peptide of the *apf* gene. In pAFβ900-IL22, the mouse IL-22 gene was fused to the sequence encoding the last 231 amino acids of the proteinase P protein (PrtP) of *L. paracasei* BL23 for covalent surface anchoring of the IL-22 on the cell wall.

The main difference between these two plasmids and our previous pAF plasmid series is that the genes encoding chloramphenicol and ampicillin are present instead of a gene encoding erythromycin. Ampicillin is used for selection of *E. coli* transformants and chloramphenicol for selection of *Lactobacillus* transformants.

### Expression of mouse IL-22 in *Lactobacillus*

**Fig. 2:**
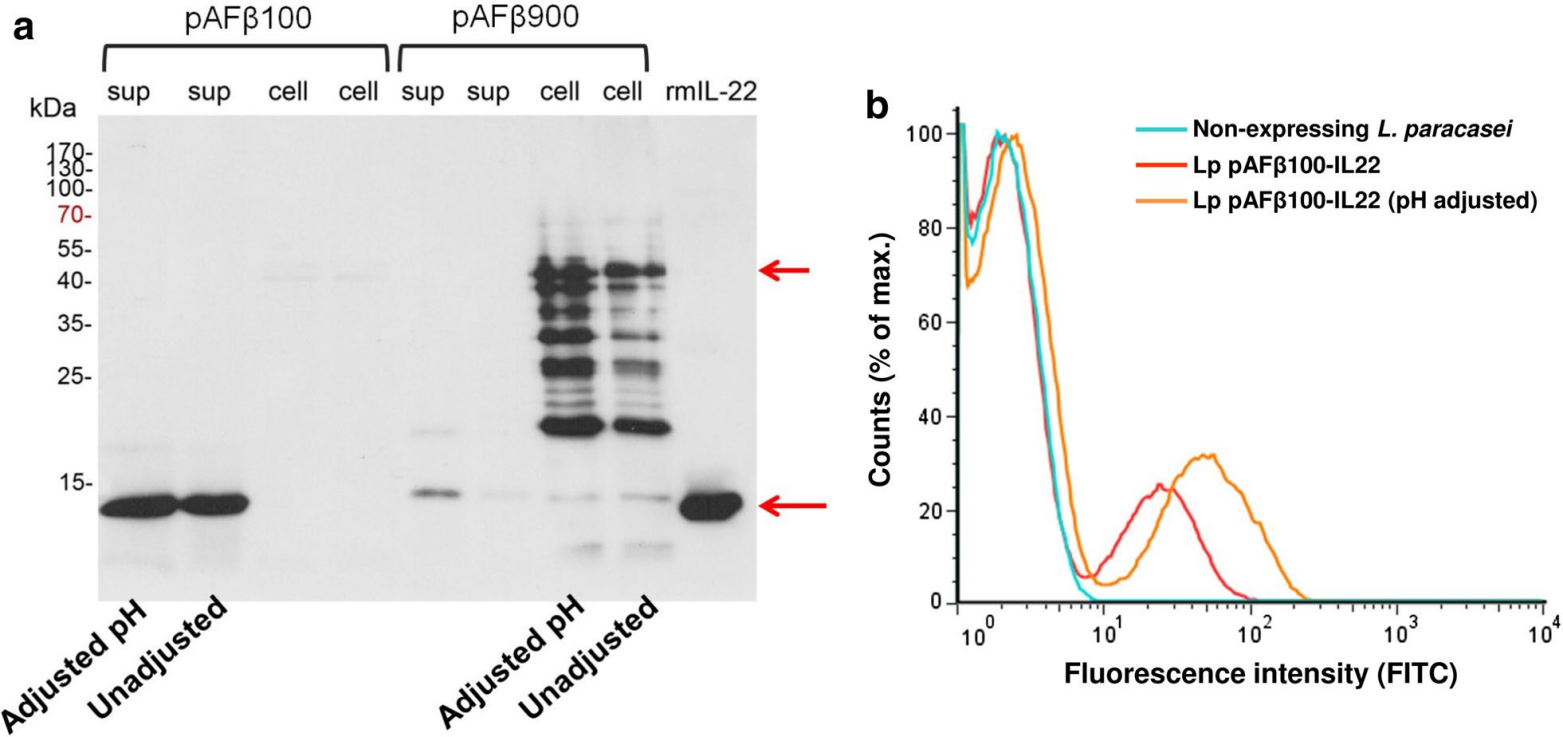
Expression and surface display of mouse IL-22 by *L. paracasei* BL23. **a** Detection of mouse IL-22 in the supernatant (sup) or cell pellet (cell) of Lp pAFβ100-IL22 (secreted-IL-22) and Lp pAFβ900-IL22 (anchored IL-22) by Western blot. **b** Surface display of anchored IL-22 (Lp pAFβ900-IL22) by flow cytometry. For both methods, the initial pH of culture MRS medium was either adjusted to pH 8.5 or not adjusted (pH 6.3)

Expression of mouse IL-22 from both the Lp pAFβ100-IL22 and Lp pAFβ900-IL22 strains was verified by Western blot analysis of the supernatant and cell pellet of the *Lactobacillus* culture (Fig. 2a). The secreted mouse IL-22 was detected only in the culture supernatant, whereas the mouse IL-22 anchored fusion protein (41.8 kDa) was detected mainly in the cell extract (Fig. 2a). The *Lactobacillus* secreted IL-22 (MW 17.2 kDa) migrate at a similar rate as the commercial IL-22 (MW 16.5 kDa) on the gel. Some IL-22 was found in the supernatant fraction of the lactobacilli expressing the anchored construct, which is likely to be either due to inefficient anchoring of IL-22 or saturation of anchoring sites. Additional bands observed for the anchored IL-22 probably result from degradation of the recombinant protein inside the bacteria.

The display of anchored mouse IL-22 on the surface of Lp pAFβ900-IL22 strain was determined by flow cytometry (Fig. 2b). It is worth noting that around 80% of bacteria were not stained by flow cytometry (Fig. 2b), indicating that surface display of IL-22 can be only detected in 20% of Lp pAFβ900-IL22.

The non-stained population of anchored construct in flow cytometry has been observed in our previous work (˂24%) but not to such a high level [24]. A large population of non-stained lactobacilli was more recently observed when we expressed other cytokines (unpublished data). Meanwhile, these strains were also growing very slow in the liquid MRS medium with antibiotic. It raises the concern about plasmid loss during the growth. To evaluate whether this non-stained population is due to the plasmid loss, the bacterial culture (at OD_600_ = 1.0) of IL-22 expressing lactobacilli were diluted and plated on MRS plates with or without chloramphenicol. The plasmid loss rate was 60% for the secreted IL-22 strain (Lp pAFβ100-IL22) and 75% for the anchored IL-22 strain (Lp pAFβ900-IL22), which is corresponding to the flow cytometric result of surface IL-22 display.

### Effect of pH on production of IL-22 by *Lactobacillus*

IL-22 belongs to the IL-10 superfamily of cytokines and shares structural similarity and 22% sequence homology to mouse IL-10 [9]. Since mouse IL-10 is a highly acid-sensitive cytokine and reported to be subjected to breakdown when the pH of the culture medium drop to pH 5.5 [36], we evaluated if mouse IL-22 was also sensitive to low pH caused by lactic acid produced by *Lactobacillus*. When grown in MRS medium, *Lactobacillus* acidifies the growth medium from 6.3 to pH 4.5. Therefore, the initial pH of the culture medium was increased to 8.5, generating a final pH around 6.0 after 5 h of growth. The difference of IL-22 production between pH adjusted and non-adjusted culture was evaluated by Western blot, ELISA and flow cytometry. In both Western blot (Fig. 2a) and ELISA, we observed only a slight reduction of IL-22 in the supernatant of Lp pAFβ100-IL22 when the initial pH was not adjusted (approximately 0.3-fold lower than pH adjusted one detected by ELISA). Flow cytometric analysis (Fig. 2b) demonstrated a twofold reduction in IL-22 expression when the Lp pAFβ900-IL22 was grown in pH non-adjusted MRS. Therefore, the effect of pH for IL-22 production is not as crucial as for IL-10.

### Recombinant mouse IL-22 expressed by *Lactobacillus* is biologically active

Mouse IL-22 shares 79% amino acid sequence identity with human IL-22 and recombinant mouse lL-22 was previously shown to induce human IL-10 secretion in Colo 205 cells in a dose-dependent manner [37]. It is a frequently used method to verify the bioactivity of commercial IL-22.

**Fig. 3:**
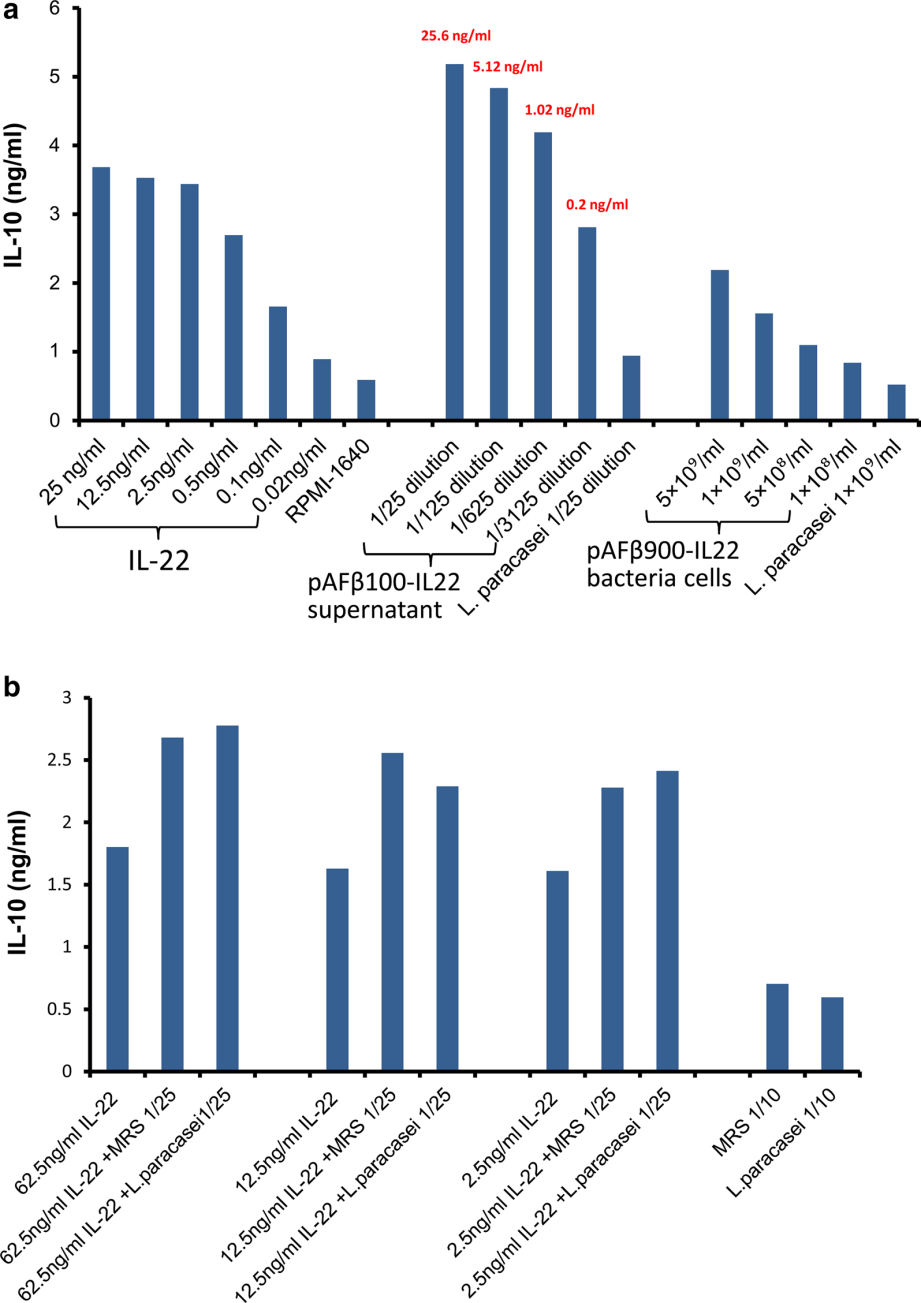
IL-10 stimulation by *Lactobacillus*-produced IL-22. **a** Dilutions of mouse recombinant IL-22, Lp pAFβ100-IL22 culture supernatant and Lp pAFβ900-IL22 bacterial cells stimulate the secretion of IL-10 in Colo 205 cells. The concentration of IL-22 in different supernatant dilutions is marked in *red*. Results are representative of three independent experiments. **b** Different concentrations of recombinant IL-22 had been spiked with either MRS medium or wild type *L. paracasei* culture supernatant

As shown in Fig. 3a, both the culture supernatant of Lp pAFβ100-IL22 and the UV treated Lp pAFβ900-IL22 bacteria could significantly stimulate IL-10 production from Colo 205 cells in a dose-dependent manner, suggesting that *Lactobacillus* is producing bioactive mouse IL-22.

As reported in the commercial IL-22 datasheet, we also demonstrated that the recombinant mouse IL-22 stimulated IL-10 secretion in Colo 205 cell in a dose-dependent manner, while the IL-10 secretion tended to plateau off when higher concentrations of IL-22 were applied (Fig. 3a). The supernatant of *Lactobacillus* secreting IL-22 demonstrates the same trend as the commercial recombinant mouse IL-22 in stimulating IL-10 secretion, however, induced even higher amounts of IL-10 secretion.

In order to identify the factors that might be enhancing the stimulation by the *Lactobacillus* supernatant, another experiment was performed, in which commercial recombinant mouse IL-22 was added to MRS or the supernatant from the wild type *Lactobacillus* strain (Fig. 3b). The induction of IL-10 was still dependent on the presence of active IL-22 but was independent of *Lactobacillus* growth, suggesting that some unknown component in the MRS medium in combination with IL-22 stimulates the IL-10 production from the Colo 205 cell line. Hence, lactobacilli expressed mouse IL-22 and commercial recombinant mouse IL-22 showed similar bioactivity in vitro.

Intraperitoneal injection of 4 µg IL-22 (1.5 × 10^14^ molecules) was previously shown to enhance the recovery of ISCs, increase epithelial regeneration and reduce intestinal pathology and mortality from graft-versus-host disease [19]. According to the quantitative Western blot results (Additional file 1: Figure S1), the concentration of IL-22 in the supernatant of Lp pAFβ100-IL22 was 0.64 μg/ml which correspond to 2.2 × 10^13^ molecules/ml at a bacterial density of approximately 2 × 10^8^ cfu/ml. The theoretical maximum number of molecule anchored on the surface of lactobacilli is estimated to be around 2 × 10^3^–6 × 10^3^ molecules/bacteria [23, 31].

If we test our *Lactobacillus* strains in the GVHD mice model in future, the mice treated with *Lactobacillus* expressing IL-22 could maximum receive daily doses of approximately 10^13^ molecules. This amount might not sufficient in order to reach the ISC compartment at the bottom of the crypt of the intestinal epithelium. Furthermore, plasmid loss was observed during growth in the culture medium and it is expected that such plasmid loss will increase in absence of antibiotic selective pressure in vivo. Further experiments should be done to improve the IL-22 delivery by integrating the expression cassette into the *Lactobacillus* chromosome and thereby stabilizing its expression. Expression of IL-22 in a mouse intestinal *Lactobacillus* strain could potentially increase the residency time of the recombinant *Lactobacillus* and contact of the bacteria with the epithelium, thereby improving the delivery of IL-22 in the ISC compartment and thus the therapeutic effect. We need to determine the best conditions for future treatment in this context, such as dose and frequency of administration of recombinant *Lactobacillus* or combined treatment with antibiotic.

## Conclusions

We have previously focused on expressing antibody fragments by lactobacilli to combat viral or bacterial infections in the gastrointestinal tract. In the present study, we have expanded our *Lactobacillus* delivery system for cytokine (IL-22) delivery. We could show that IL-22 can be expressed by lactobacilli in both the secreted form and anchored to the cell wall, and retains its bioactivity in vitro. Improving IL-22 delivery of our *Lactobacillus* system might thus be required before evaluating its future therapeutic effect.

## Additional file

**Additional file 1: Figure S1.** Concentration of IL-22 as quantified by Western blot densitometry. Dilutions of pure commercial IL-22 (5–0.625 ng) and culture supernatant were loaded onto the gel and detected by Western blot. The concentration of expressed protein in the supernatant was calculated with reference to the standard curve consisting of known concentrations of the purified reference protein. **Table S1.** Primers and synthetic genes used in this study.
