# Supplementary material for: Lactobacillus delivery of bioactive interleukin-22
Source: Microb Cell Fact. 2017 Aug 23;16:148. doi: 10.1186/s12934-017-0762-1 (PMC5567760; doi:10.1186/s12934-017-0762-1)
Supplement: Supplementary file 1 — Additional file 1: Figure S1. Concentration of IL-22 as quantified by Western blot densitometry. Dilutions of pure commercial IL-22 (5–0.625 ng) and culture supernatant were loaded onto the gel and detected by Western blot. The concentration of expressed protein in the supernatant was calculated with reference to the standard curve consisting of known concentrations of the purified reference protein. Table S1. Primers and synthetic genes used in this study. [file 12934_2017_762_MOESM1_ESM.pdf]

Supplemental data

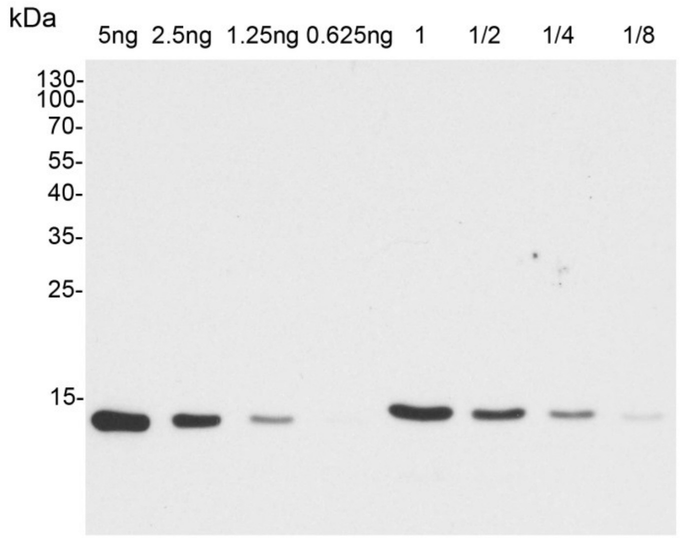

Figure S1 Concentration of IL-22 as quantified by Western blot densitometry. Dilutions of pure commercial IL-22 (5 ng to 0.625 ng) and culture supernatant were loaded onto the gel and detected by Western blot. The concentration of expressed protein in the supernatant was calculated with reference to the standard curve consisting of known concentrations of the purified reference protein.

Table S1 Primers and synthetic genes used in this study.

Primer or gene	Sequence (5'-3')	Features
APFSalS	CGCGTCGACGGATAAGGCAGAATAATGGAATAA	Forward, SalI
APFBamAS	CGGGATCCTTCTACAATAGCAGCTTGAGCAGT	Reverse, BamHI
APFSacS	CGAGCTCTCAACTGTAAGTGGTTCAGAAGCT	Forward, SacI
EcoAS2	CGGAATTCCTTGAACCGTTTGTGGTGTCGTTT	Forward, EcoRI
prtPSacS	CGGAGCTCAAGAAGACTTCGCTGCTTAACCAGT	Forward, SacI
prtPSacAS	CGGAGCTCCTATTCTTCACGTTGTTTCCGTT	Reverse, SacI
IL-22 synthetic gene	GGATCCTTGCCTGTCAACACCCGCTGTAAGCTGGAGGTCTCAAACTTTCAACAACCGTATATTGTGAATCGGACTTTTATGCTGGCCAAGGAAGCGAGCTTAGCCGATAATAACACTGATGTTCGTTTGATTGGGGAAAAGTTATTTCGGGGCGTGTCCGCGAAGGATCAGTGTTATTTGATGAAGCAAGTGCTGAACTTTACCCTGGAAGATGTCTTGTTACCGCAGTCGGATCGGTTTCAGCCATATATGCAAGAGGTTGTGCCGTTTCTGACGAAATTGTCAAACCAATTGAGTTCGTGCCATATTTCTGGCGATGATCAGAATATTCAAAAGAACGTCCGTCGGCTGAAAGAAACCGTTAAGAAATTGGGCGAGTCTGGTGAGATTAAGGCGATTGGGGAACTGGATTTGTTATTTATGTCACTGCGGAATGCTTGCGTTTAAGAGCTC	For cloning in pAFβ100-IL22. A similar gene without stop codon (TAA) was cloned in pAFβ900-IL22.
